# Potential role of N-carbamoyl glutamate in biosynthesis of arginine and its significance in production of ruminant animals

**DOI:** 10.1186/2049-1891-4-16

**Published:** 2013-04-10

**Authors:** Bahram Chacher, Hongyun Liu, Diming Wang, Jianxin Liu

**Affiliations:** 1 Institute of Dairy Science, College of Animal Sciences, Zhejiang University, 866 Yuhangtang Road, Hangzhou 310058, P R China; 2 MOE Key Laboratory of Molecular Animal Nutrition, Zhejiang University, Hangzhou 310058, People's Republic of China

**Keywords:** Arginine, Lactation, N-cabamoyl glutamate, Nitrogen utilization, Pregnancy, Ruminant animals

## Abstract

Arginine (ARG) exerts many beneficial effects on animal body and enhanced angiogenesis, lactogenesis, which finally leads to the improvement in nitrogen (N) metabolism, reproduction, lactation, immunity and growth. Unfortunately, unprotected ARG will be degraded in the rumen and its price is high, thus feeding rumen-protected ARG seems to be uneconomical. Alternatively, N-carbamoyl glutamate (NCG) is structural analogue of N-acetyl glutamate, cofactor of cabamoyl phosphate synthetase1, is lower in rumen degradation compared to ARG. Additionally, rumen epithelial and duodenal cells have potentially utilized the NCG for ureagenesis. Supplementation of NCG to high yielding dairy cows increased plasma concentration of ARG and nitric oxide, decreased the plasma ammonia N and improved lactation performance and N utilization. Supplementation of NCG enhanced pregnancy rates in rats, improved litter size and fetal survival rate, thereby improved the reproductive performance of sows. Oral NCG supplementation increases plasma ARG and somatotropin levels, and increased growth rate and muscle protein synthesis in nursing piglets. The NCG is potential a relatively cheaper source of feed additive to offer vital compensation over oral administration of ARG, resulting in improved ruminant animal health and production. In this article, we reviewed the mechanism of ARG biosynthesis by NCG and their significance in growth, reproduction, milk production and N utilization in ruminant animals.

## Introduction

Supply of all essential and non-essential amino acids (AA) to animals at systemic level is of great importance to assure the demands of AA for both maintenance and production. Arginine (ARG) is one of the most versatile AA, which serves as a precursor for synthesis of urea, nitric oxide (NO) and polyamines and regulates key metabolic pathways that are critical to health, growth, reproduction and homeostasis of the animals [1]. Despite the fact that ruminant can synthesize it, ARG is normally considered to be essential, because *denovo* synthesis is not sufficient to meet the requirement particularly during the early stages of growth or for high level of production [2] Moreover, ARG is well recognized for functioning of ureagenesis and ammonia detoxification [3]. Generally, high performance animals are offered diets rich in protein, which could impair the ureagenesis and increase the plasma ammonia concentration, resulting in infertility, decreased milk production, increased nitrogen (N) excretion in urine and feces contributing to environmental pollution. Therefore, supplementation of targeted nutrients could improve fertility, milk production and N utilization in high-producing animals by decreasing plasma ammonia load through efficient regulation of urea cycle.

In the previous studies it has been showed that infusion of ARG could improve the N metabolism in heifers [4] and milk production in cows [5]. Parenteral administration of ARG in ewes decreased embryonic loss, increased lamb birth weight, and improved survival rate of fetal lamb to term in prolific ewes [6,7]. However, ARG was rapidly degraded in rumen [8], and parental administration of ARG to farm animal is not a practical approach, while feeding of rumen-protected ARG seems to be uneconomical. N-cabamoyl glutamate (NCG) is a structural analogue of N-acetyl glutamate (NAG) [9], and is low in rumen degradation [8]. Supplementation of NCG increased the endogenous synthesis of plasma ARG in piglets [10]. Oba et al. [11] confirmed that rumen epithelial and duodenal cells could utilize the NCG for urea synthesis. Moreover, feeding NCG to animals seemed to be more beneficial than ARG, because oral administration of NCG rather than ARG entered the systemic circulation completely in adults [12]. Additionally, NCG with low costs is available from chemical synthesis [13]. These advantages indicate that NCG is a possibly cheap source of feed additive that improve the ruminant production and benefit the dairy producers.

Limited information is available on function of NCG in ruminant animals. In this article, we reviewed the mechanism of ARG biosynthesis in which the NCG is involved and their potential function in reproduction, growth, lactation and N utilization in ruminant animals.

### Arginine biosynthesis in which NCG is involved in ruminant animals

The ARG is an integral constituent of urea cycle, a major pathway for urea synthesis and ammonia detoxification. The biosynthesis of urea is initiated in mitochondria of heptocycte and intestinal cells by the action of cabamoyl phosphate synthetase 1 (CPS I), whose activity is stimulated by NAG. The ARG is a direct allsoteric activator of NAG synthase, a mitochondrial enzyme converting glutamate and acetyl coenzyme A to NAG [14,15]. On the other hand, NCG is active but biologically stable structural analogue of NAG [9] that is co-factor of the first rate-limiting enzyme CPS1 [16], while CPS1 remains inactive in the absence of NAG [15]. The NAG is readily hydrolyzed *in vivo*, while the NCG is stable in both *in vivo* and *in vitro* situation and resistant to degradation by aminoacylase. Moreover, NCG can cross the mitochondrial membrane to enter inside [17]. Therefore, ARG can be synthesized endogenously from glutamate via pyrroline-5-carboxylate (P5C), ornithine, citrulline, and argininosuccinate. The P5C synthase and NAG synthase are the two key regulatory enzymes in the intestinal citrulline synthesis [16]. Thus, NCG is also called the ARG raiser. Generally, AAs are required for optimal growth, reproduction, lactation and maintenance, but the quantification of proteins and AAs are difficult due to complex rumen metabolism.

In our previous study [8], the proportion of ARG and NCG degradation in rumen fluid for 24 h was 100.0% and 17.8%, respectively. Addition of NCG and ARG increased *in vitro* gas production and acetate to propionate ratio and diminished microbial protein mass compared with the control. Rapid degradation of ARG in rumen is a nutritionally wasteful process. Thus, ARG should be spared from rumen degradation, while NCG could be fed to ruminant without need for coating [8]. Furthermore, NCG is much cheaper than ARG [13]. In addition, NCG has variable advantages over ARG. The NCG does not interfere with intestinal absorption of dietary tryptophan and basic AA, and may result in a balanced ARG during the suckling period due to constant activation of intestinal synthesis of citrulline by NCG. Low dose of NCG is effective in activation of both P5C synthase and CPS-I. Our preliminary results of feeding 20 and 30 g/d NCG to high yielding dairy cows indicated an increased plasma ARG concentration [18]. Therefore, NCG is potential feed additive to rise plasma ARG concentration and to enhance ruminant performance.

### Significance of NCG in reproduction

#### Conception rate and early embryo loss

Reproductive efficiency in dairy cows is decreasing worldwide. The increased in milk production per cow is one of contributing factors. Compared with traditional dairy system, the cows in modern intensive farms have longer intervals to first ovulation, higher incidence of anestrous, abnormal luteal phases, higher twinning rates and greater embryonic loss [19]. High yielding dairy cows are usually fed diets rich in crude protein, which consequently elevated plasma urea N concentration and was associated with decreased fertility in dairy cows because the nitrogenous waste products such as ammonia and urea N are considered to be toxic in bovine gametes and/or embryos and easily cause reproductive inefficiency in dairy cows [20]. Whereas, high concentration of plasma urea N in early lactation elevated the ammonia N and urea N concentrations in both follicular fluid of pre-ovulatory follicles and uterine fluid during the luteal phase of the estrous cycle [21].

Supplementation of NCG can increase endogenous synthesis of ARG, resulting in increases plasma ARG concentration [9]. It has been indicated that feeding NCG significantly decreased the plasma ammonia N contents and restore ureagesis in both human and piglets [9,22]. Therefore, conception rates of high yielding cows that fed high protein diet can be improved by preventing ammonia toxicity to embryo through NCG supplementation.

The early development of bovine embryo relies on uterine secretions until implantation, while deviations in the uterine environment can be detrimental to development and survival of embryo [23]. In the early embryo development, leukemia inhibitory factor plays an essential role [24]. One of the initial events during embryo implantation is adhesion of trophoblast cells to glycoproteins in the extra cellular matrix of the uterine epithelium (fibronectin, vitronectin and laminin). Leukemia inhibitory factor promoted the adhesion of extravillous trophoblast to fibronectin, vitronectin and laminin during the first trimester of pregnancy [25]. Dietary ARG supplementation could improve embryo implantation in pregnant rats and exhibited an increase in embryonic survival and litter size [26].

Interestingly, supplementation of NCG enhanced pregnancy rates in rats through activation of the phosphatidylinositol 3-kinase/protein kinase B/mammalian target of rapamycin signaling pathway [27]. The NO and polyamines are key regulators of angiogenesis and embryogenesis, as well as placental and fetal growth [28]. High milk production and improved conception rates are important to dairy producers. Collectively, these studies provide evidence that reproductive efficiency in ruminant can be enhanced via supplementation of targeted nutrients such as ARG or NCG.

#### On fetus during late pregnancy

Normally, ewes can give birth to one to three lambs. Multiple pregnancies increase the risk for fetal and neonatal death in ewes, while uterine capacity is a major factor inhibiting fetal survival and growth in mammals [29]. This maternal constraint is particularly evident in pregnant ewes with multiple fetuses, since nutrients and space to nurture all fetuses can not be adequately met, and consequently birth weights and survival rates of lambs are reduced resulting in intrauterine growth restriction (IUGR) [29,30]. However, no treatments have been currently used to prevent IUGR in sheep from gestating multiple fetuses.

As a common precursor for synthesis of NO and polyamines, ARG is crucial for placental angiogenesis and growth in mammals [31]. Therefore, alterations in the ARG-NO and polyamine pathways are contributed to impaired utero-placental blood flow and IUGR in animals. There is evidence that ARG enhanced fetal growth in ewes by serving IUGR carrying quadruplets [7]. Proper developments of the placenta are critical for a successful pregnancy [32], while vasculogenesis and angiogenesis are critical for both proper placental function and normal embryonic/fetal growth and development [32,33].

Generally, the angiogenic process is initiated by growth factors such as vascular endothelial growth factor (VEGF) and placental growth factor 1 [33]. The VEGF is considered to be the most important factor promoting the differentiation of mesenchymal cells in the villous core into hemangioblastic stem cells and is mostly known to regulate the processes of vasculogenesis and angiogenesis [32]. As a potent endothelial survival factor, VEGF induces vasodilation and facilitates blood flow by increased NO production, and is considered as potential promoter of endothelial permeability [34]. Through elevated endothelial NO synthase in placental surface vessels and increased nutrients supply to fetus, NCG may improve litter size and fetal survival [35]. It has been demonstrated that dietary supplementation of ARG or NCG affect microRNAs (miR-15b, miR-222) targeting VEGFA and endothelial NO synthase gene expressions in umbilical vein, consequently regulate the function and volume of the umbilical vein, provide more nutrients and oxygen from the maternal to the fetus tissue [36]. Therefore, supplementation of NCG during late pregnancy to ruminant animals is an effective strategy for fetal development and survival.

### Effect of NCG on growth and cell proliferation

#### Body homeostasis by increasing brown adipose tissue

Healthy newborn calves, lambs and kids provide foundation for profitable dairy enterprises. Unfortunately, neonatal mortality remains a significant problem worldwide. Many newborn, particularly lambs and kids, die during winter season due to body heat loss rather than disease. The rate of heat loss is affected by several factors, the most important one of which is to maintain body temperature when livestock exposed to cold environments where oxidation of nutrients and body reserves will be used to generate heat. In sheep, fetal brown adipose tissues, main fat reserves oxidized for heat, start to be generated after mid-gestation [37,38]. Flyn and Wu [39] demonstrated that endogenous synthesis of ARG played an important role to maintain ARG homeostasis in neonatal pig and weaned piglets. Decreased maternal obesity and multiple advantages of fetal growth (organ and tissues development and fetal brown adipose tissue enhancement) were also induced by supplementation of ARG [40]. Therefore, supplementation of NCG to sheep and goat could be best way to increased plasma ARG concentration in specific period of pregnancy during winter season could maintain the homeostasis of neonate and prevent the mortality and consequently improve productivity and profitability of ruminant animals.

### Weaned weight and growth

Minimizing the stress during weaning ensures the calves to continue production process. Before weaning, calves are highly susceptible to gastrointestinal disorders, especially diarrhea. Weaning is a stressful event in the calf's lifetime with alterations in behavior [41], and immune function [42]. Dietary supplementation of NCG could enhance intestinal growth and mRNA abundance of heat shock protein, prevent intestinal dysfunction and ameliorated stress induced by weaning, and improve growth performance in weanling pig [43]. Additionally, ARG is the most abundant N carrier in tissue protein and may be responsible for maximal growth of young mammals [44]. The plasma ARG and somatotropin levels were increased by oral supplementation of NCG, and growth rate and muscle protein synthesis was improved in nursing piglets [10]. Supplementation of NCG increased the proliferating cell nuclear antigen mRNA and induced cell growth and proliferation in intestinal mucosa, thus improved intestinal mucosa morphology in weaning piglets [45]. Weaning weight and plasma ARG concentrations were greater when calves received an ARG supplemented ration [46]. It is reported that dietary supplementation with 1.0% glutamate prevented weaning-induced villus atrophy in jejunum of weanling piglets, and combined supplementation of glutamate and NCG had favorable effect on intestinal epithelium cell proliferation and prevented intestinal mucosa dysfunction [47]. It is inferred that feeding NCG to calves and lambs during weaning stage could decreases the stress, enhance immunity and weight gain, and consequently improve animal productity and profitability.

### Significance of NCG in lactation and N utilization

#### Lactation performance

Managing high producing cows has always been challenge for dairy farmers and nutritionists. As a precursor of NO, ARG has caused great interest due to the potential role of NO in regulating mammary tissue nutrient perfusion [48]. Increased amount of nutrients available to mammary glands is critical to enhance AA uptake for milk production [49]. Thus, the number of mammary cells and the amount of available nutrients are the dominant factors for milk production.

It has been showed that ARG increased milk production in cattle and growth hormone in heifers [4,5]. However, mechanism of improved milk production induced by ARG needs to be elucidated. On other hand, high yielding animals need diet containing high protein for maximum production that consequently causes the ammonia toxicity and decreased feed intake [50], whereas Lobley et al. [51] reported that ureagenesis may compete with other process that alters AA required for milk synthesis. It is well recognized that ARG plays a key role in ureagenesis [3]. In our preliminary study [18] feeding NCG to high yielding dairy cows significantly decreased the plasma ammonia N and increased NO concentration compared to control. Yield of milk and milk protein and contents of milk protein and lactose were also increased.

### Nitrogen utilization

Dairy animals are inefficient in N conversion, and only 25%-30% of dietary N can be converted into milk [52]. Formulation of low N diet can be efficient approach to reduce N excretion, but decreased milk production [53], particularly in high yielding cows. Alternatively, N utilization can be improved by increased conversion rate of intake protein [54], increased urea recycling in gut and liver and optimized AA balance [11]. Oba et al. [11] confirmed that rumen epithelial cells and duodenal cells had the capacity to utilize the NCG for urea N recycling if CPS1 enzyme activity is stimulated. Thus, urea synthesis in gut tissues could provide a potential target to decrease ammonia absorption and improve N utilization in ruminant [55]. In our current study, supplementation of 20 g/d NCG to high yielding dairy cows significantly decreased the urea N excretion in milk, plasma and urine, while the N utilization tended to be improved [18], indicating that feeding NCG to high yielding dairy cows in early to mid lactation is an effective strategy to improved the N utilization.

## Conclusions

The NCG has been potentially substituted for ARG in non-ruminant animal. Lower rumen degradation of NCG has made it cheaper compared to ARG in ruminant industry. Supplementation of NCG to high yielding cows can increase plasma ARG concentration and milk production. The N utilization and reproductive efficiency in ruminant can be enhanced by supplementation of NCG, indicating that NCG may be a novel feed additives for ruminant that could not only substitute of ARG, but has some beneficial effect on N utilization. Further studies should be conducted to see the effect of NCG on metabolic mechanism, growth and reproductive performance of ruminant animals.

## Abbreviations

ARG: Arginine; CPSI: Carbamoyl phosphate synthetase1; IUGR: Intrauterine growth restriction; N: Nitrogen; NAG: N-acetyl glutamate; NCG: N-carbamoyl glutmate; NO: Nitric oxide; P5C: Pyrroline-5-carboxylate; VEGF: Vascular endothelial growth factor.

## Competing interests

The authors declare that they have no competing interests.

## Authors’ contributions

BC collected the information used in manuscript, and drafted and revised the manuscript. HYL collected the information and helped to draft the manuscript. DMW checked the language and grammar of the manuscript. JXL participated in concept designing the manuscript and critically revised the manuscript. All authors made substantial inputs to the review and approved the final manuscript.
